# Feasibility of magnetic resonance-guided high-intensity focused ultrasound treatment targeting distinct nodular lesions in neurofibromatosis type 1

**DOI:** 10.1093/noajnl/vdab116

**Published:** 2021-08-18

**Authors:** Caitlin Tydings, Pavel Yarmolenko, Miriam Bornhorst, Eva Dombi, John Myseros, Robert Keating, James Bost, Karun Sharma, AeRang Kim

**Affiliations:** 1 Center for Cancer and Blood Disorders, Children’s National Hospital, Washington, District of Columbia, USA; 2 Sheikh Zayed Institute for Pediatric Surgical Innovation, Children’s National Hospital, Washington, District of Columbia, USA; 3 Gilbert Neurofibromatosis Institute, Children’s National Hospital, Washington, District of Columbia, USA; 4 National Cancer Institute, Pediatric Oncology Branch, National Institutes of Health, Bethesda, Maryland, USA; 5 Department of Neurosurgery, Children’s National Hospital, Washington, District of Columbia, USA; 6 Department of Biostatistics and Study Methodology, Children’s Research Institute, Washington, District of Columbia, USA; 7 Department of Radiology, Children’s National Hospital, Washington, District of Columbia, USA

**Keywords:** distinct nodular lesions, magnetic resonance-guided high-intensity focused ultrasound, neurofibromatosis type 1, targeted therapy

## Abstract

**Background:**

Patients with Neurofibromatosis Type 1 (NF1) and plexiform neurofibromas (PN) often have radiographically diagnosed distinct nodular lesions (DNL) which can cause pain and weakness. Magnetic resonance-guided high intensity focused ultrasound (MR-HIFU) can precisely and accurately deliver heat to thermally ablate target tissue. The aim of this study is to evaluate whole-body MRIs from patients with NF1 and DNL, applying volumetrics and a consistent treatment planning approach to determine the feasibility of MR-HIFU ablation of DNL.

**Methods:**

A retrospective review of whole-body MRI scans from patients with NF1 and PN from CNH and NCI was performed. DNL are defined as lesions >3 cm, distinct from PN and lacking the “central dot” feature. Criteria for MR-HIFU thermal ablation include target location 1–8 cm from skin surface; >1 cm from visible plexus, spinal canal, bladder, bowel, physis; and ability to ablate ≥50% of lesion volume. Lesions in skull and vertebral body were excluded.

**Results:**

In 26 patients, 120 DNL were identified. The majority of DNL were located in an extremity (52.5%). Other sites included head/neck (7%), chest (13%), and abdomen/pelvis (28%). The predefined HIFU ablation criteria was not met for 47.5% of lesions (*n* = 57). The main limitation was proximity to a vital structure or organ (79%). Complete and partial HIFU ablation was feasible for 25% and 27.5% of lesions, respectively.

**Conclusion:**

Based on imaging review of lesion location, technical considerations and ability to target lesions, thermal ablation with MR-HIFU may be a feasible noninvasive alternative for symptom management in patients with NF1 and symptomatic DNL.

**Key Points**

- Symptomatic DNL can contribute to morbidity in patients with NF1.
- Some DNL are recognized as ANNUBP when biopsied with malignant potential.
- MR-HIFU may be feasible for the treatment of symptomatic DNL in patients with NF1.

**Importance of the Study**

MR-HIFU has the potential to contribute greatly to both adult and pediatric populations due to its noninvasive and nonradiative approach, eliminating many side effects associated with current standard therapies including surgery. Patients with NF1 have plexiform neurofibromas and often radiographically diagnosed distinct nodular lesions which grow out of proportion to surrounding plexiform neurofibroma, causing pain and weakness. Surgical removal for symptom management is complicated and not always feasible. MR-HIFU could provide a noninvasive alternative to surgery for the treatment of symptomatic DNL.

Magnetic resonance-guided high-intensity focused ultrasound (MR-HIFU) is a novel, noninvasive and nonionizing therapy that provides controlled and focused delivery of heat to a precise target. An external applicator called a transducer is used to focus ultrasound energy into a small volume of tissue within a specific target site seen with MRI. This energy is focused onto small regions measuring a few millimeters in size, which allows for precision and minimal potential of thermal injury to surrounding areas. Coupling with MR thermography allows for precise real-time temperature monitoring and control.^1^ MR-HIFU can apply varying degrees of hyperthermia ranging from mild hyperthermia (40–45°C) to ablation (>55°C). MR-HIFU thermal ablation leads to instantaneous cell death by coagulative necrosis.^2^

MR-HIFU has been FDA-approved for the treatment of painful bone metastases, essential tremor, tremor-dominant Parkinson’s disease, benign prostatic hyperplasia, prostate cancer, and uterine fibroids. Based on its application for these indications, HIFU is combined with either MRI or ultrasound-based imaging. Insightec’s Exablate Neuro device is the only MR-HIFU system to be approved for neurological applications.^3^ The Sonalleve HIFU system (Profound Medical) has received *Conformité Européene* (CE) marking for uterine fibroids and painful bone metastases. Recently, this system received US FDA Humanitarian Device Exemption approval for the treatment of osteoid osteoma, a benign bone tumor, in the extremities.^4,5^ MR-HIFU is being used in multiple pediatric clinical trials to treat bone and soft-tissue tumors including painful osteoid osteoma^5–7^ and desmoid tumors^8,9^ as well as other pediatric solid tumors in a variety of locations (NCT02076906, NCT02536183).

MR-HIFU ablation is an outpatient-based treatment that has been tolerated in adults with most common adverse effects being skin burns, pain and edema which are acute and reversible in the majority of cases.^1^ Limited experience in pediatrics has also proven to be safe with no major complications and adverse events that are acute and reversible.^9^ Limitations to treatment with MR-HIFU may include the ability to completely target lesions given their variability in location and significant intertwining with a nerve plexus. In order to limit potential side effects caused by off-target heating, a safety margin of 1 cm is applied to nearby critical structures and the skin surface. MR-HIFU, as a noninvasive therapy, may avoid the complications associated with surgical resection such as infection, bleeding, nerve damage, and scar formation.

In this study, we evaluate the feasibility of applying MR-HIFU to a soft tissue tumor observed in patients with the tumor predisposition syndrome Neurofibromatosis Type 1 (NF1). The hallmark lesions of NF1 are neurofibromas, a type of benign peripheral nerve sheath tumors (PNST).^10^ Some neurofibromas form large conglomerate masses along multiple nerve fascicles and branches, called plexiform neurofibromas (PN).^11^ NF1 patients are also at risk of developing malignant peripheral nerve sheath tumor (MPNST), an aggressive and treatment resistant soft tissue sarcoma. MPNSTs often originate from a pre-existing benign PNST and develop through an intermediate phase of atypical neurofibroma (ANF).^12^ The transition is characterized by genetic (*CDKN2A/B* mutation), histopathological (nuclear atypia, increased variable cellularity, disorganized architecture, and some mitotic activity), metabolic (increased PET avidity), and morphological changes (focal growth).^10,12–15^ The term atypical neurofibromatous neoplasm with uncertain biologic potential (ANNUBP) has recently been proposed by a consensus of expert pathologists to describe lesions that have histopathologic features associated with higher risk of malignant transformation.^10,12^

Distinct nodular lesions (DNL) are identified radiographically as neurofibromas that are round, well-demarcated, larger than 3 cm in longest diameter and with loss of central dot sign associated with PN.^10,15^ DNL is a descriptive term based on imaging features; however, many of these lesions are categorized as ANNUBP when biopsied, though not all ANNUBP are DNL. DNLs evolve over time, with the growth often exceeding the growth of other PNSTs within the same patient.^15^ This can cause pain, disfigurement and neurological dysfunction. Surgical resection may be considered for symptomatic DNLs, or lesions with concerning atypia. Local recurrence is rare, even with suboptimal margins, suggesting that progression towards MPNST can be prevented.^16^ On the other hand, surgery may carry significant risks such as bleeding and neurologic deficits.^17^

Given the role of MR-HIFU in successfully ablating several types of soft tissue tumors, we hypothesize that MR-HIFU ablation may be a feasible alternative treatment for DNL in patients with NF to alleviate symptoms and potentially prevent the transformation to MPNST. The objective of this study is to evaluate whole-body MRIs of NF1 patients with identified DNL, applying volumetrics and consistent treatment planning to determine the feasibility of MR-HIFU ablation of DNL.

## Materials and Methods

### Study Subjects

The Gilbert Family Neurofibromatosis Institute at Children’s National Hospital (CNH), Washington, DC sees approximately 750 pediatric and young adult patients with NF1 yearly. The Pediatric Oncology Branch (POB) at National Cancer Institute (NCI), Bethesda, MD is an international referral center and leading NF center with a large patient population. This is a retrospective review of MRI images from NF1 patients with plexiform neurofibroma(s) who had whole-body MRIs.

Patients from CNH were enrolled on clinical trial NCT03820778 evaluating the use of whole-body MRI for identifying ANNUBP in patients with NF1. These patients had high plexiform tumor burden defined as ≥1 PN that was >3 cm in diameter. Patients from NCI were enrolled on clinical trial NCT00924196, a natural history study of patients with NF1. Patients selected from this study were those with known DNL identified on MRI and representing a cohort to cover a range of sizes, location and anatomy. Patients enrolled met the NIH diagnostic criteria for NF1. MRI scans occurred between 2010 and 2019. Twenty-six patients were identified and included in this study. Only one whole-body MRI per patient was evaluated without prior or subsequent MRIs for comparison due to availability of whole-body MRIs per patient on study. The protocols were approved by respective institutional review boards. Informed consent was obtained from all patients/guardians.

### DNL and MR-HIFU Criteria

DNL are defined as lesions ≥3 cm in largest dimension, distinct from plexiform neurofibroma and lacking the “central dot” feature.^10,15,18^ MR-HIFU ablation criteria is based on a consensus of safety measures felt to reduce risk in clinical trials.^19,20^ Also, while different MR-HIFU systems exist with varying design specifications to treat targets in different locations, for the purpose of this study, we will be basing criteria for treatment on the clinical MR-HIFU system available at CNH (Profound Sonalleve V2; Profound Medical Inc., Mississauga, Ontario, Canada).

Lesions were considered either targetable (greater than 50% ablation feasible) or not targetable. Criteria for a targetable lesion included targets >1 cm and <8 cm from skin surface with a beam path that was not obstructed by intervening bone or air-tissue interfaces such as lung and bowel. Target lesions also had to be >1 cm from major neurovascular bundle, plexus, spinal canal, bladder, bowel, and open physis.

Lesions were considered not targetable if located in the head and neck due to proximity to critical structures and limited institutional experience. Lesions in the chest and abdominal cavities were considered not targetable due to the impact of motion from respiration and peristalsis on ability to perform accurate temperature monitoring, as well as acoustic shadowing, or inability to treat through gas-containing structures such as lungs and bowel. Exceptions included axillary lesions that extend from the upper extremity into chest cavity and one posterior lesion in the superior aspect of chest cavity due to minimal impact from respiration. The partial ablation cut off of 50% was decided upon by a consensus of our team of experts as a benchmark of what we could measure to say a lesion was accessible by HIFU.

### DNL Identification and Measurement

Osirix software (Pixmeo SARL; v10.0.0) was used for all evaluations and measurements. Whole-body MRIs were closely examined, identifying all lesions meeting criteria for DNL. Lesions were reviewed and confirmed to be DNL by a team of radiologists and oncologists. Digital calipers were used to measure all lesions in both axial and coronal planes. The median longest diameter of all lesions was calculated.

### Treatment Planning for MR-HIFU Thermal Ablation

**Figure 1.**
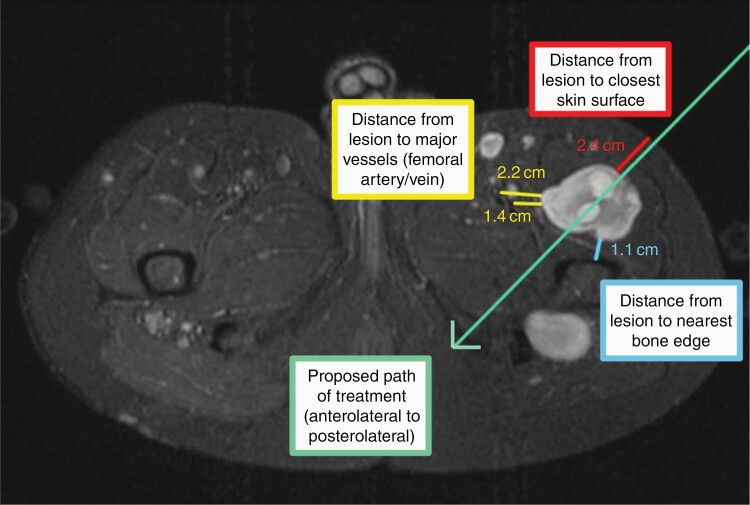
Treatment planning of DNL in left proximal thigh showing proposed treatment path and distances from skin, major neurovascular bundle and bone.

For lesions which met predefined criteria and threshold to be considered targetable, volumetric analysis and treatment planning were performed. Volumetric analysis was performed using Osirix software. Total tumor volume (TTV) was measured by outlining the DNL margins on each axial MRI slice showing tumor. Each lesion was individually evaluated for complete or partial ablation using digital calipers to measure the closest distance from edge of tumor to skin surface, major neurovascular bundle and nearby gas-containing organs. A line of treatment, representing the proposed treatment beam, was placed over axial planes in direction of desired treatment approach (Figure 1). Approach was based on best angle and beam path to avoid obstruction, limit effect on nearby structures and effectively treat the largest percentage of tumor. Treatment planning was reviewed by a team including interventional radiology, engineering, oncology, and neurosurgery.

Lesions meeting criteria for MR-HIFU and greater than 1cm from skin surface, major neurovascular bundle and nearby organ on all axial slices had a planned treatment volume (PTV) of 100% and were considered capable of undergoing complete ablation. Lesions less than 1 cm from any of these structures underwent a second volumetric analysis. Initial TTV was adjusted to create a new tumor border allowing for greater than 1 cm distance to skin surface, major neurovascular bundle and nearby organ. The remaining volume was calculated to be PTV. The percent treatable tumor was calculated as 100 × PTV/TTV. Lesions with percent treatable tumor >50% were considered capable of undergoing partial ablation >50%.

### Statistical Analysis

In this retrospective review, feasibility of MR-HIFU for the treatment of DNL is summarized descriptively. Categorical variables were reported as percentages and when compared using the Chi-squared test. Continuous measures were reported as means (SD) or Median (IQR) and assessed with the Wilcoxon Rank Sum Test or the Kruskal–Wallis Test.

## Results

### Patient Characteristics

Twenty-six patients with Neurofibromatosis Type 1 and plexiform neurofibromas followed with whole-body MRI were identified from NIH (*n* = 21 patients) and CNH (*n* = 5 patients). Eighteen patients were male and 8 were female with a median age of 21 years (range 10–41 years). Among 26 patients, 120 DNL were identified with locations including head/neck (*n* = 8), chest/abdomen/pelvis (*n* = 50), upper extremities (*n* = 16), and lower extremities (*n* = 48). There were a median of 4 DNL per patient (range 1–19) and a median of 50% of lesions per patient were targetable.

### MR-HIFU Targetability

**Figure 2.**
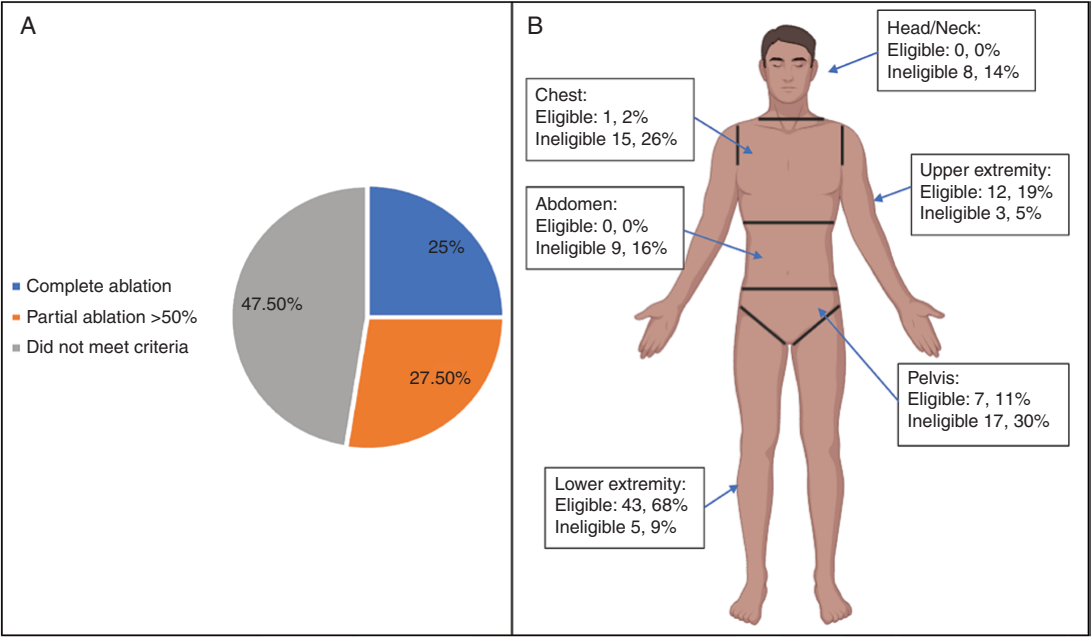
(A) Percentage of 120 total DNL able to undergo complete ablation, partial ablation or did not meet criteria for ablation. (B) DNL eligibility for ablation based on location and ability to be >50% ablated. Percentages based on 63 eligible DNL and 57 ineligible DNL.

After excluding the nontargetable lesions located in the head, neck, chest, and abdomen (*n* = 52), 57% of lesions (*n* = 68) were targetable for MR-HIFU based solely on location. Following, volumetric analysis and treatment planning, 30 lesions (25%) met criteria for complete ablation and 33 lesions (27.5%) met criteria for partial >50% ablation (Figure 2a). An additional 5 lesions were included in those not meeting criteria for ablation (total *n* = 57 lesions, 47.5%) after volumetric analysis and treatment planning revealed <50% ablation was possible. Lesions that did not meet criteria for ablation were ineligible based on location and could not be ablated >50% following volumetric analysis. Figure 2b displays percentage of eligible and ineligible DNL based on their location.

Of the 30 DNL meeting criteria for complete ablation (Table 1), 3% were located in upper extremity (*n* = 1), 20% in pelvis (*n* = 6) and 77% in lower extremity (*n* = 23). Median TTV 11.9 mm^3^ (range 2.5–99.2 mm^3^) for all 30 lesions, 34 mm^3^ for upper extremity lesion, 11.9 mm^3^ for lower extremity lesions, and 14.4 mm^3^ for pelvic lesions. All lesions had a PTV of 100%.

**Table 1.** Complete Ablation DNL Characteristics and Volumetrics

Location	n = 30	%	Median Total Tumor Volume (mm^3^)	Range (mm^3^)
Upper extremities	1	3	34	34
Lower extremities	23	77	11.9	2.5–99.2
Pelvis	6	20	14.4	11.8–31.6
Total	30		11.9	2.5–99.2

Thirty-three lesions met criteria for partial ablation (Table 2) with a median TTV of 28.4 mm^3^ (range 3.8–530mm^3^). Following treatment planning and revised volumetric analysis, median PTV was 24.9 mm^3^ and median percent PTV was 88.3% (51.8–98.8%). Thirty-three percent (*n* = 11) of lesions were in the upper extremity with a median TTV of 34.4 mm^3^ (12.3–89.7 mm^3^), PTV of 25.2 mm^3^ and percent PTV of 79.6%. Sixty-one percent (*n* = 20) of lesions were located in lower extremities with a median TTV of 22.7 mm^3^ (3.8–530 mm^3^), PTV of 22.5 mm^3^ and percent PTV of 93.4%. Six percent of lesions were located in the chest (*n* = 1) and pelvis (*n* = 1). The chest lesion had a median TTV of 13.2 mm^3^, PTV of 12 mm^3^ and percent PTV of 99.1%, and the pelvic lesion had a median TTV of 24.3 mm^3^, PTV of 18.4 mm^3^, and percent PTV of 75.9%.

**Table 2.** Partial Ablation >50% DNL Characteristics and Volumetrics

Location	n=33	Median Total Tumor Volume (mm^3^)	Range (mm^3^)	Median Planned Treatment Volume (mm^3^)	Median Percent Planned Treatment Volume (%)
Upper extremity	11	34.4	12.3–89.7	25.2	79.6
Lower extremity	20	22.7	3.8–530	22.5	93.4
Chest	1	13.2	13.2	12	99.1
Pelvis	1	24.3	24.3	18.4	75.9
Total	33	28.4	3.8–530	24.9 (2.9–497.5)	88.3 (51.8–98.8)

The median (IQR) treatable tumor volumes for complete (11.9 mm^3^ (8.1–31.6)) and partial (24.9 mm^3^ [12.0–59.9]) were statistically significantly different (*P* = .049) utilizing the Wilcoxon Rank Sum Test. Location of tumor as it relates to ability to be completely or partially ablated was not found to be statistically significant.

### MR-HIFU Limitations

Limitations to complete ablation but still meeting criteria for partial ablation (*n* = 33) included lesions <1 cm from skin (*n* = 11, 33%), <1 cm from neurovascular bundle (*n* = 18, 55%), and <1 cm from lung (*n* = 4, 12%). Of the 57 lesions that were not considered targetable, limitations included proximity to skull (*n* = 8, 14%), spine (*n* = 11, 19.3%), bowel (*n* = 24, 42.1%), skin (*n* = 4, 7%), lung (*n* = 7, 12.3%), neurovascular bundle (*n* = 3, 5.3%) (Table 3).

**Table 3.** Limitations Excluding DNL from Ablation

Limitation	n = 57	%
Lesions near skull	8	14
Paraspinal	11	19.3
Bowel	24	42.1
<1 cm from skin	4	7
Lung	7	12.3
<1 cm from neurovascular bundle	3	5.3

## Discussion

MR-HIFU is a novel, noninvasive and nonradiative therapy that can provide precise heating and tissue death while sparing normal, surrounding tissue. It has proven to be very tolerable in both adult and pediatric populations, eliminating many risks associated with invasive and ionizing alternative therapies. Neurologic applications are an exciting new frontier, particularly for intracranial pathologies leading to movement and psychiatric disorders.^21,22^ However, its role in neurofibromas has never before been described.

Through longitudinal analysis of whole-body MRI in NF1 patients, DNL have been identified which have different growth patterns from surrounding PN and can cause patients pain and neurologic symptoms including weakness. Surgery to remove symptomatic neurofibromas has its limitations in feasibility and resultant morbidities, particularly intraoperative blood loss and long term neurologic deficits.^23^ MEK inhibitors have been proposed as treatment for neurofibromas but was interestingly found to be less effective in DNL than in PN.^24^ This retrospective review of whole-body MRI showed that thermal ablation of DNL with MR-HIFU may be feasible alternative to medical therapy for or surgical resection of symptomatic DNL.

Of the 120 identified DNL in 26 patients from NIH and CNH, 68 lesions (57%) were felt to be targetable for MR-HIFU treatment based on location alone. Lesions located in the head, neck, and abdomen were not targetable due to limited use of the current MR-HIFU system near the skull and technical difficulties of lesions located in the abdomen due to motion from bowel peristalsis and acoustic shadowing from bowel. Lesions located in the chest were largely not considered targetable due to proximity to lung and impact of movement from respiration. An exception to this included lesions that extended from the axilla, labeled as upper extremity lesions, and one smaller lesion that was located in the superior aspect of the chest, posterior to lungs. These exceptions were made due to minimal impact of motion from respiration and target beam could be diverted away from critical structures including lungs.

Following treatment planning and volumetric analysis, 30 lesions met criteria for complete ablation and were predominantly located in the lower extremities (77%). Of the 33 lesions that met criteria for partial ablation, the median percent PTV was 88.3% with a range of 51.8%–98.8% suggesting that despite inability to perform complete ablation, most lesions could undergo near-complete ablation. Not surprisingly, given origins of DNL from plexiforms, the most common limitation in 55% of these lesions was proximity to a major neurovascular bundle. Smaller tumors were more likely to be completely ablated than partially ablated. Patients are drawn from large referral centers at CNH and NCI where whole-body MR is performed and patients are more likely to have a higher tumor burden which likely biases our study population.

**Figure 3.**
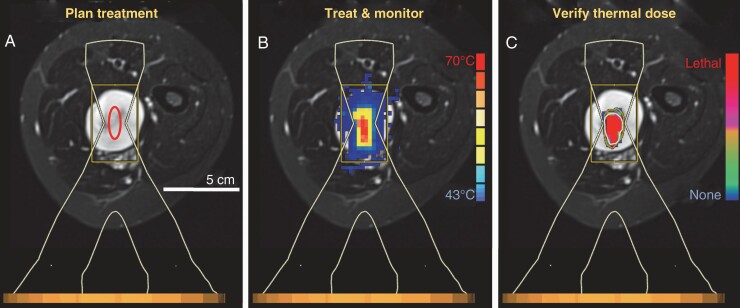
Potential workflow of DNL ablation with MR-HIFU. A) An ablation is planned by placing a treatment cell (8 mm diameter cell used in this case) over the lesion, using the HIFU beam outline to avoid critical neurovascular bundles and nearby bone. B) Multiplanar MR thermometry is used to monitor the ablation in real time. C) Thermal dose is monitored throughout the sonication and its values at the end of the sonication are used to display a volume of complete coagulative necrosis. The ablation duration is <20 s, followed by an approximate cooling period of 3 minutes during which the target and surrounding tissues return to a steady state temperature. Additional treatment cells are then placed and sonicated to cover the rest of the targeted DNL lesion. The MR thermometry and thermal dose overlays are provided here only as examples from an unrelated sonication, to demonstrate the approximate extent of heating with a single sonication.

The use of MR-HIFU in the treatment of symptomatic neurofibromas is a novel application. In order to prioritize safety with early technology and risking under-ablation, 50% was chosen as a cut off for partial ablation. While 50% was a metric we used, there was a spectrum of treatment volumes with the majority of lesions able to undergo near-complete ablation. We felt it was prudent to first figure out how MR-HIFU is going to work for this indication, with the goal of near-complete (80%–100%) ablation with subsequent experiments. It is unclear if there is potential for rebound growth or other manifestations with partial treatment. It is not known how MR-HIFU may react with neurofibromas based on their unique density, water content and vascularity which vary from other tissue types already studied. Because of this, the amount of energy required for tissue ablation is unclear. Based on prior experience, a maximum treatment volume of 120cc per treatment would be feasible. The number of DNL per patient ranged from 1–19 with a median of 4. Multiple lesions could be ablated in the same treatment session as long as patient positioning would not significantly prolong the time under anesthesia. Figure 3 demonstrates the proposed treatment workflow of a DNL located in the thigh using a lateral positioning.

Limitations to this retrospective study of whole-body MRI include inability to control patient positioning and MRI sequences. Treatment planning was performed on provided imaging that had varying quality and suboptimal positioning that may not reflect desired position to target a particular lesion. Volumetrics may also have been affected by patient positioning as the skin surface may be artificially closer to lesions, especially in extremities, on surfaces in contact with table. MR-HIFU complications include the need for general anesthesia in most cases to allow for optimal positioning and the potential for long treatment times, possibly requiring multiple treatments to completely ablate larger lesions. Given whole-body MRIs were taken from clinical trials, these MRIs represented a moment in time and not necessarily the most recent MRI. Also, due to this, comments could not be made on tumor changes or growth.

Treatment planning required maintaining a distance of 1cm from major neurovascular bundles; however, smaller vessels or nerves in closer proximity to the treatment zone may not be spared from thermal damage. This may result in neurologic damage that could manifest as neuropathic pain or loss of function of innervated areas. Another complication, particularly with superficial lesions include skin burns which can be mitigated by allowing for skin cooling between each treatment. It is unclear what the complication rate would be compared to that of surgery. Given surgery does not require a 1cm margin, larger PTV could theoretically be achieved with surgical techniques. However, there may be exceptions to certain limitations placed on MR-HIFU in this study given safety criteria applied is based on our group’s experience with benign and malignant solid tumors. For example, it is possible that with nerve stimulation testing, a closer margin may be achieved.

In conclusion, MR-HIFU may be feasible in the treatment of DNL in patients with NF1. While greater than 50% of DNL identified could be at least 50% ablated, it is unclear what percentage of the lesion needs to be ablated to relieve or improve symptoms. One interesting, potential application of MR-HIFU is the treatment of ANNUBP as an alternative to surgical resection in order to prevent growth and transformation of ANNUBP to MPNST. Future studies are required to understand the safety and effect of MR-HIFU treatment of neurofibromas. We are developing a Phase 0 clinical trial of NF1 patients with biopsy-proven ANNUBP planning to undergo surgical resection who will be treated with MR-HIFU ablation prior to surgical resection. This would allow us to assess the safety and feasibility of HIFU ablation for ANNUBP in patients with NF1 and evaluate treatment effects on tissue histology.
